# Improving crop productivity and nitrogen use efficiency using sulfur and zinc-coated urea: A review

**DOI:** 10.3389/fpls.2022.942384

**Published:** 2022-10-14

**Authors:** Ayesha Mustafa, Fareeha Athar, Imran Khan, Muhammad Umer Chattha, Muhammad Nawaz, Adnan Noor Shah, Athar Mahmood, Maria Batool, Muhammad Talha Aslam, Mariusz Jaremko, Nader R. Abdelsalam, Rehab Y. Ghareeb, Muhammad Umair Hassan

**Affiliations:** ^1^Department of Agronomy, University of Agriculture, Faisalabad, Pakistan; ^2^Department of Agricultural Engineering, Khwaja Fareed University of Engineering and Information Technology, Rahim Yar Khan, Pakistan; ^3^College of Plant Science and Technology, Huazhong Agricultural University, Wuhan, China; ^4^Division of Biological and Environmental Sciences and Engineering, Smart-Health Initiative and Red Sea Research Center, King Abdullah University of Science and Technology, Thuwal, Saudi Arabia; ^5^Agricultural Botany Department, Faculty of Agriculture (Saba Basha), Alexandria University, Alexandria, Egypt; ^6^Plant Protection and Biomolecular Diagnosis Department, Arid Lands Cultivation Research Institute, City of Scientific Research and Technological Applications, New Borg El Arab, Egypt; ^7^Research Center Ecological Sciences, Jiangxi Agricultural University, Nanchang, China

**Keywords:** coated urea, growth, photosynthesis, nitrogen use effiency, nitrogen loss

## Abstract

Nitrogen (N) is an important macro-nutrient required for crop production and is considered an important commodity for agricultural systems. Urea is a vital source of N that is used widely across the globe to meet crop N requirements. However, N applied in the form of urea is mostly lost in soil, posing serious economic and environmental issues. Therefore, different approaches such as the application of urea coated with different substances are used worldwide to reduce N losses. Urea coating is considered an imperative approach to enhance crop production and reduce the corresponding nitrogen losses along with its impact on the environment. In addition, given the serious food security challenges in meeting the current and future demands for food, the best agricultural management strategy to enhance food production have led to methods that involve coating urea with different nutrients such as sulfur (S) and zinc (Zn). Coated urea has a slow-release mechanism and remains in the soil for a longer period to meet the demand of crop plants and increases nitrogen use efficiency, growth, yield, and grain quality. These nutrient-coated urea reduce nitrogen losses (volatilization, leaching, and N_2_O) and save the environment from degradation. Sulfur and zinc-coated urea also reduce nutrient deficiencies and have synergetic effects with other macro and micronutrients in the crop. This study discusses the dynamics of sulfur and zinc-coated urea in soil, their impact on crop production, nitrogen use efficiency (NUE), the residual and toxic effects of coated urea, and the constraints of adopting coated fertilizers. Additionally, we also shed light on agronomic and molecular approaches to enhance NUE for better crop productivity to meet food security challenges.

## Introduction

The rate at which the global population is growing is posing a serious threat to food security (He et al., 2021). By 2050, the population is predicted to exceed 9.2 billion people (Silva, 2018), resulting in a 60–102% rise in food consumption (Elferink and Schierhorn, 2016). Currently, agricultural nitrogen utilization has already exceeded its “sustainable” level, calling for efficient nitrogen usage; however, that alone does not address the need for increasing agricultural production required by a growing population (He et al., 2021). On the other hand, continuous water shortage, labor scarcity, nutrient mining, and loss of soil fertility are significantly reducing agricultural production across the globe (Shivay et al., 2019; Mohammed et al., 2020).

Nitrogen plays a pivotal role in many physiological processes, grain quality, and biomass production (Anas et al., 2020). It plays a vital role in forming chlorophyll, proteides and proteins, and other essential compounds like plant hormones; however, plants are inefficient in the acquisition and utilization of applied nitrogen (Tiong et al., 2021). Additionally, the application of conventional urea is also not so efficient leading to excessive nitrogen loss from the soil. Therefore, farmers use enormous amounts of N to boost crop productivity which is not just undesirable but leads to many environmental problems (Elferink and Schierhorn, 2016). Excessive use of nitrogen (N) fertilizers causes soil acidification and groundwater pollution by water eutrophication, which also increases greenhouse gas emissions (Mohanty et al., 2020). Therefore, there is an urgent need to adopt an efficient N management approach such as nutrient-coated urea which increases nitrogen use efficiency (NUE), crop quality, and yield (Prasad and Hobbs, 2018).

**Figure 1:**
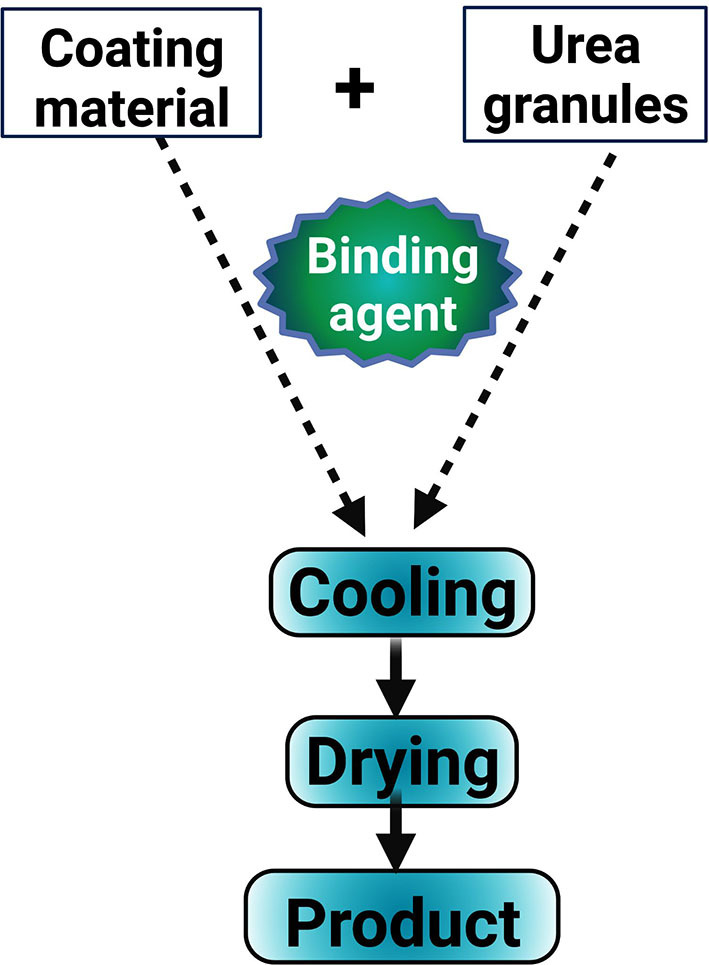
Graphical presentation of coated urea preparation. The urea is mixed with matter to be coated and allowed to cool and dry to obtain coated urea.

The coating technology is designed for the slow release of the nutritional content of fertilizers and synchronization of their release rate with the nutritional demand of the plants. The characteristics of the gradual release of nitrogen contents can be physically imparted to the urea by coating its granules with various materials which delay its dissolution rate (Naz and Sulaiman, 2016; Ullah et al., 2020; Badshah et al., 2021; Al-Nemi et al., 2022). Coated fertilizer (Figure 1) is believed to be the best solution under the current scenario to deliver nutrients to crops for enhancing their productivity, reducing nutrient losses, and minimizing the subsequent impact on the environment (Shivay et al., 2019; Shah et al., 2022). Nutrient-coated urea behaves as a slow-release fertilizer as the thin nutrient layer that is applied over the urea granules hydrolyzes slowly and remains in the soil for a longer period, which in turn improves crop productivity and efficient nutrient use. Moreover, coated urea also improves nitrogen availability along with other macro and micronutrients that are vital for crop growth and development (Khan et al., 2015). Different coated fertilizers are currently available in local markets, but in this review, our main focus is on sulfur and zinc-coated urea.

Sulfur is a fundamental element required for nitrogen metabolism and protein synthesis and is mandatory for many enzymatic and metabolic activities including plant defense mechanism systems against biotic or abiotic stress (Khan et al., 2014; Yu et al., 2018; Tavanti et al., 2021). The application of S-coated urea substantially improves crop growth, development, and yield compared to uncoated urea (Xin et al., 2017; Shivay et al., 2019). Sulfur-coated urea (SCU) boosts root growth by increasing its diameter and length which further helps in increasing nutrient uptake (Timilsena et al., 2015), and plays a part in dry matter assimilation (Haseeb-ur-Rehman et al., 2022). It also improved the availability of nitrogen (N), phosphorus (P), potassium (K), and zinc (Zn) (Najafian and Zahedifar, 2015), and improved chlorophyll content and photosynthetic efficiency (Awais et al., 2015). SCU augments the gradual release of nitrogen leading to a reduction in nitrogen leaching and volatilization, thereby increasing nitrogen use efficiency (Ke et al., 2018).

**Figure 2:**
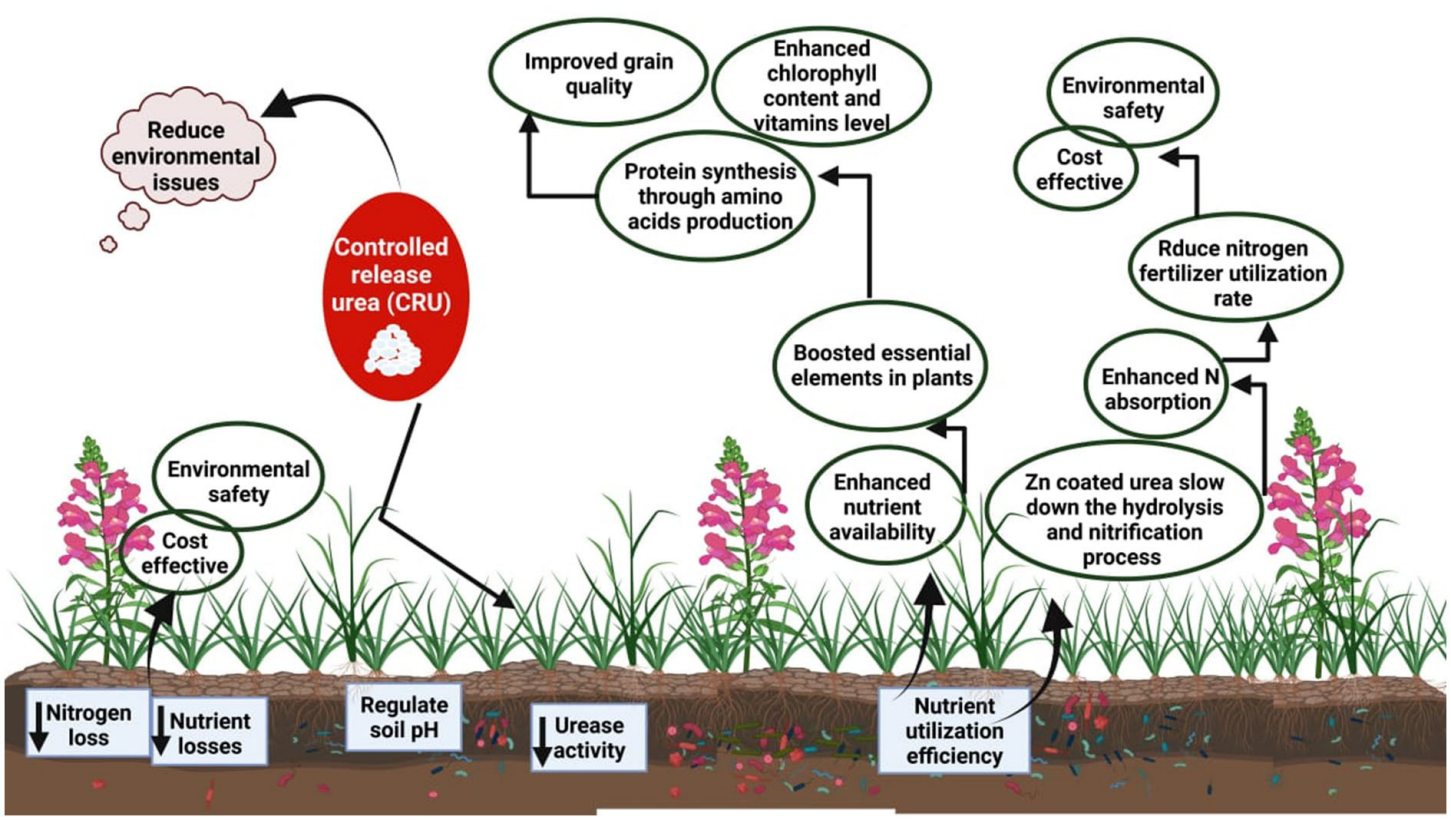
Benefits of coated urea. Urea coating reduces leaching and volatilization losses, improves grain yield, reduces the costs of fertilizer application, and increases nutrient absorption and nutrient availability in the soil.

Zinc is an essential element for optimum plant growth and plays an important function in metabolism. It is necessary for the proper functioning of various physiological functions in plants, including photosynthesis and sugar generation, fertilization and seed production, growth regulation, and disease resistance (Hassan et al., 2020). Zinc stimulates enzymes involved in the creation of certain proteins. It aids the plant's ability to survive low temperatures by assisting in the creation of chlorophyll and certain carbohydrates, as well as the conversion of starch to sugar (Chattha et al., 2017; Hassan et al., 2019; Suganya et al., 2020; Hassan M. U. et al., 2021). Zinc-coated urea is a source of sufficient nutrients (Zn and N) that meet the initial requirements of growing plants (Qureshi et al., 2021; Beig et al., 2022). By boosting plant physiology and metabolism, zinc-coated urea (ZnCU) has the potential to provide a long-term enhancement in agricultural productivity (Figure 2) and quality (Ain et al., 2020; Hassan M. et al., 2021; Rehman et al., 2021). Zinc-coated urea increases zinc concentration in rice and wheat grains, resulting in higher crop quality (Irshad et al., 2021).

The controlled release mechanism of nitrogenous fertilizers prevents N losses in plants, and the urea coated with compatible compounds is beneficial for the slow distribution of nitrogen and enhancement of NUE in plants. In this review, we focused on nutrient-coated urea, crop performance, their dynamics in soil, toxicity, and the deficiency of zinc and sulfur in plants, exploring agronomic and molecular approaches interlinked with sulfur and zinc-coated urea that enhance NUE and crop production.

## Role of zinc and sulfur in plant biology

Sulfur and zinc are imperative nutrients required for plant growth and development. Sulfur contributes to the production of various metabolites, which in turn improve plant performance in terms of growth and productivity (Gigolashvili and Kopriva, 2014). Moreover, sulfur also plays a significant role in the electron transport chain and improves plant resistance to stressed conditions (Hell et al., 2010). Sulfur and nitrogen have the same assimilation pathway and S-containing metabolites are crucial to the photosynthetic process (Mazid et al., 2011). Zinc plays a significant role in physiological functions and it is necessary for the maintenance of protein structure functions, synthesis, enzyme structure, gene expression, energy production, carbohydrate metabolism, auxin metabolism, Krebs cycle, and photosynthesis (Nadeem et al., 2020). Moreover, Zn also protects against abiotic stresses and substantially improves crop productivity and quality (Nadeem et al., 2020).

## Effect of zinc and sulfur-coated urea on photosynthesis

When sulfur absorption is reduced from 2,500 to around 500 (g g^−1^) dry weight; the rate of photosynthetic CO_2_ uptake from intact leaves, cyclic and non-cyclic photo-phosphorylation of isolated chloroplasts is significantly disrupted. The photosynthetic efficiency in plants is drastically reduced under S deficiency as sulfur is involved in the production of chlorophyll content (Figure 2) which has a direct impact on the rate of photosynthesis (Abadie and Tcherkez, 2019). Sulfur also protects the plant from oxidative stress and thereby improves the plant's performance under stressed conditions. Sulfur-containing proteins play a significant role in photosynthesis, carbon metabolism, and subsequent production of assimilates (Hooghe et al., 2013). The chlorophyll content can be reduced up to 39–65% in the event of S deficiency (Padhi et al., 2013) due to the accumulation of sugars in plant roots being increased by sulfur uptake (Abadie and Tcherkez, 2019). Sulfur inhibits several metabolic activities, including the bruising of chlorophyll membranes, the loss of pigments from cells, and the breakdown of chlorophyll, which results in diminished photosynthesis (Padhi et al., 2013).

Chlorophyll content, photosynthesis, and respiration rate along with carbonic anhydrase activity increase with zinc application. A small amount of zinc concentration of 13–14 g/g dry weight is required for optimum photosynthesis and chlorophyll production (Raza et al., 2018). Zinc is also involved in the production of chlorophyll content by regulating the amounts of specific nutrients in the cytoplasm (Aravind and Prasad, 2004, 2005). Zinc deficiency in plants results in disrupted photosynthesis due to changes in chloroplast structure and pigments (Kosesakal and Unal, 2009). It sustains the chlorophyll levels and is vital for maintaining chloroplast structure in plants (Chen et al., 2007).

## Effect of zinc and sulfur-coated urea on protein synthesis

Methionine, homocysteine, cysteine, and taurine are the four main sulfur-containing amino acids. Amino acids regulate protein metabolism, and protein synthesis is significantly altered when there is a deficiency of sulfur-containing amino acids (David et al., 2016). They also regulate nutrient metabolism (Shigi et al., 2021). Methionine, a component of many proteins, is important in DNA and RNA synthesis and serves as a methyl group donor. Several sulfur-containing nucleosides consist of the derivatives of 2-thiocytidine (s2C), 2-thiouridine (s2U), 4-thiouridine (s4U), and 2-methylthioadenosine (ms2A), which ensure sulfur availability in each biosynthetic channel (Shigi, 2014). Intracellular antioxidants glutathione and N-acetyl cysteine are produced by sulfur amino acids which are involved in the antioxidant defense mechanism (Colovic et al., 2018). Cysteine also forms a disulfide bond which plays a pivotal role in protein-folding pathways and protein structure (Brosnan and Brosnan, 2006). Sulfur increases the uptake of P (phosphorus) and N (nitrogen) during seed formation; moreover, it is an integral part of nucleic acid and protein synthesis (Shigi et al., 2021).

Zinc fingers (small protein structured motif) allow enzymes to act as a template for the production of a specific protein by transcribing a second genetic division from DNA to RNA. They have a wide range of functions, including DNA recognition, RNA packaging, transcriptional activation, apoptosis regulation, protein folding and assembly, and lipid binding. In addition, Zn catalyzes numerous enzymatic actions and helps keep protein sub-domains folded (McDonald et al., 2000). It is essential for the tetrahedral bonding of particular genes for the transcription of protein. As a result, a loop of 11-13 A.A. (amino acids) forms DNA-metalloproteins, which are then used as a DNA expression in the protein translation process with the help of Zn (McDonald et al., 2000). Zinc is a component of ribosomes and is responsible for protein synthesis. McDonald et al. (2000) found ribosomal disintegration in the absence of Zn. Moreover, Zn deficiency is the most common cause of protein synthesis inhibition, resulting in lower amounts of ribonucleic (RNA) acid and reduced plant growth (Krishna et al., 2020).

## Effect of zinc and sulfur-coated urea on growth and development

Sulfur is an indispensable element for plant growth and development. It has been identified as a key nutrient that improves crop growth and quality (Table 1). Enough sulfur is required for plant functions and is critical for optimal crop performance (Marschner, 2012). It is taken up by the plant in the form of sulfate, which is absorbed by the roots or shoots. Sulfur deficiency lowers the concentration and activity of the ribulose-I,5-bisphosphate carboxylase/oxygenase (Rubisco) enzyme resulting in a substantial reduction in photosynthesis (Makino, 2003) which causes a significant decrease in plant growth, development and grain production (Chan et al., 2019). Sulfur protects plants from diseases in their reproductive stage—the period when plants are more vulnerable to sulfur deficiency. Sulfur deficiency reduces protein quality and many secondary metabolites involved in plant developmental mechanisms (Saleem et al., 2019).

**Table 1:** Effect of sulfur-coated urea on growth, physiology, and yield attributes.

**Crop**	**Rate of sulfur-coated urea**	**Major effects**	**References**
Wheat	130 kg/ha	Application of S-coated urea improved crop growth and yield traits	Adil et al., 2021
Rice	270 kg/ha	The application of S-coated urea improved the growth, amylose, amylopectin, and starch contents and teste value	Hai-yan et al., 2018
Rice	160 kg N/ha	S-coated urea improved chlorophyll contents, grain weight, and grain yield	Haseeb-ur-Rehman et al., 2022
Wheat	130 kg N/ha	S-coated urea increased hlorophyll contents, net leaf photosynthetic rate and leaf area index, and grain yield	Gafoor et al., 2021
Wheat	140 kg/ha	S-coated urea increased the grain yield and NUE of wheat crop	Malakouti et al., 2008
Rice	60 kg/ha	S-coated urea improved dry matter production, grain yield and NUE of rice crop	Khan et al., 2015
Soybean and mungbean	60 kg N/ha	Coated urea improved the growth, yield traits, and grain production of soybean and mungbean crops	Essa et al., 2021

Zinc is an essential component of several enzymes, which are responsible for a variety of metabolic reactions in all crops. It is also necessary to produce tryptophan, a precursor to IAA and a growth-promoting substance (Cabot et al., 2019). It is involved in cell division, cell proliferation, pollen production, and disease resistance, thus, influencing crop yield (Vadlamodi et al., 2020). Zinc deficiency can delay plant growth, shorten crop maturation time, produce spikelet sterility, and diminish harvest product quality (Suganya et al., 2020). Zinc in plants promotes leaf development, flowering, and production of gibberellin and trehalose 6-phosphate (signaling chemicals), as well as regulating the vegetative growth stage (Gonzalez et al., 2012). Pollen generating capacity, anther, and pollen grain size along with their viability are decreased under low Zn supply (Rudani et al., 2018).

## Effect of zinc and sulfur-coated urea on assimilate partitioning

Accumulation of photosynthates in grains is the end product of photosynthetic activity. Carbon dioxide assimilation, sugar generation, nitrogen fixation (Table 2), and protein formation all require sulfur (Ali et al., 2013). Sulfur positively influences dry matter accumulation because it involves photosynthates partitioning from source to sink, resulting in a significant improvement in grain production (Rani et al., 2009). It also helps in glucose translocation to the reproductive segments of plants (Sahoo et al., 2018). Sulfur deficiency in oilseeds during early growth stages limits plant growth resulting in lowering biomass production. Ahmad et al. (2005) found that plants uptake and store sulfur and use it in their developmental and physiological processes; besides, sulfur has a synergistic effect with N, P, and K, which increase the biological yield in crops (Chen et al., 2010).

**Table 2:** Effect of zinc-coated urea on growth, physiology, and yield attributes.

**Crop**	**Rate of zinc-coated urea**	**Major effects**	**References**
Rice	120 kg/ha	Zn-coated urea increased the physiological use efficiency and grain yield of rice crop	Shivay and Prasad, 2012
Rice and wheat	120 kg/ha	Zn-coated urea increased the partial factor productivity, agronomic efficiency, apparent recovery, and grain yield of rice and wheat crops	Shivay and Prasad, 2012
Wheat	180 kg/ha	Zn-coated urea effectively enhanced the growth, growth, photosynthesis, physiological and yield, parameters of wheat	Nazir et al., 2021
Pasture	217 kg/ha	Coated urea significantly improved the pasture production and nutrient uptake	Junejo et al., 2011
Rice	120 kg/ha	Coated urea increased the rice productivity, kernel weight, and kernel quality of rice crop	Irshad et al., 2021

Zinc plays a chief role in assimilating portioning and its moderate applications (3.3–4.4 kg ha^−1^) can increase nutrient uptake and biomass production (Shrestha et al., 2020). Zinc deficiency reduces sucrose content in sugar, beet, and maize (Rengel and Pearson, 1997) and decreases assimilate partitioning toward reproductive parts, resulting in a decrease in crop output. Zinc insufficiency also affects carbohydrate transport and grain sucrose availability (Song et al., 2015), in addition to lowering carbonic anhydrase activity, which prevents CO_2_/HCO_3_ transport in the leaf mesophyll for photosynthetic CO_2_ fixation and in turn impairs photosynthetic rate and lowers biomass accumulation (Siddiqui et al., 2015). Toxic Zn levels hinder root sinking force and reduce assimilate translation by inhibiting root development and slightly expanding shoot growth (Gupta et al., 2016). The flow of photo assimilates, especially Zn, into grains (the sink) is governed by the source-sink connection throughout the grain-filling stage, and Zn-coated urea also stimulates Zn flow from source to sink (Chen et al., 2020). Sink capacity and strength are determined by the number and size of grains, as well as their ability to absorb Zn from source organs. The concentration of Zn in grains sinking presently varies between 20 and 35 mg/kg globally (Chen et al., 2020).

## Dynamics of nutrient-coated urea in soil

To cope with the food security challenges, farmers have utilized extensive application of chemical fertilizers to increase crop yields in recent years (Qiao et al., 2016). Urea is a basic synthetic fertilizer that is regarded as a primary source of plant nutrition (Trenkel, 2010). Conventional urea has less NUE because most of the nitrogen is wasted in denitrification, volatilization, and leaching, which is harmful to the environment and has led to agro-ecological issues (Naz et al., 2014). Therefore, the best nitrogen management technique needs to reduce nitrogen losses in the agriculture sector and maximize the efficient use of nitrogen for sustainable agricultural development (Lu et al., 2010). Controlled release urea (CRU) or nutrient-coated urea is less soluble due to the coating on the urea granules that progressively release (Table 3) the fertilizer and makes it ideal for crop synchronization (Trenkel, 2010). It increases crop production while reducing nutrient losses to the environment. Controlled release fertilizers (CRFs) have been extensively explored to make them safer and more cost-effective. They keep nutrients in the soil for the longest possible time and increase NUE by 20–30% (Cong et al., 2010). Nutrient-coated fertilizers also improve water efficiency, soil water retention capacity, soil texture and structure, and ultimately, soil quality (Fashola et al., 2006). As urease inhibitors, the usage of environmentally acceptable coated urea and micronutrients can be advantageous as a two-in-one solution. They can temporarily reduce urease activity in the soil and function as a vital nutrient for plants and the soil. These materials are easily available, biodegradable, and inexpensive. The use of natural materials as an adhesive maintains nitrogen and micronutrients on the microsite together. As a result, the urease inhibitory and acidifying properties of natural materials control the rise in pH and urea concentration on the applied soil surface. To minimize nitrogen loss (Table 4) and improve urea efficiency, urea can be coated with sulfur, urease inhibitors, or other biodegradable compounds (Shaviv, 2001). Urease inhibitors (agrotain) are chemicals that limit the action of the urease enzyme in soil and slow down the urea hydrolysis process. The efficacy of these coated fertilizers varies significantly depending on land use, soil type, and soil moisture regime (Bolan et al., 2004). Sulfur-coated (SCU) has a projected release timing ranging between 45 and 180 d; however, the greatest impact on SCU's N release comes from temperature as the rate of diffusion doubles for about every 10°C increase (Hopkins, 2020). Fertilizer-related concerns such as leaf burning, water pollution, and eutrophication are minimized. Slow nutrient release reduces runoff and leaching losses by keeping available nutrient concentrations in soil solution at a lower level (Table 5). Alkaline soils with a lower pH provide improved nutrient absorption. Because both sulfur and urea contribute to raising soil acidity (dropping soil pH), applying sulfur-coated urea is likely to enhance soil acidity (Trenkel, 2010). Throughout all phases of plant growth, the nutrient release meets the crop's nutritional requirements. Using CRFs and SRFs at appropriate moments can meet the timely plant nutrient demand requirements, increase fertilizer usage efficiency, and reduce environmental issues.

**Table 3:** Profile of various coated urea in soil.

**Coated urea**	**Function**	**Release mechanism**	**Longevity**	**References**
Sulfur-Coated urea	Nitrification inhibitor	Microbial activity	45–180 days	Baboo and Manager, 2016
Zinc-Coated urea	Urease inhibitor	Diffusion	20–140 days	Moosa et al., 2021
Neem-Coated urea	Nitrification inhibitor	Diffusion	15–90 days	Shilpha et al., 2017
Polymer-Coated urea	Control release urea	Temperature, Soil moisture	10–75 days	Osman, 2017
Carbon-Coated urea	Urease inhibitor	Cation exchange capacity (CEC), Acid buffering capacity	10–120 days	Jia et al., 2021
Boron-Coated urea	Urease inhibitor	Diffusion	10–100 days	Shivay et al., 2019
Castor oil-coated urea	Nitrification inhibitor	Temperature	10–50 days	Zhao et al., 2021
Garlic oil-coated urea	Urease inhibitor	Soil moisture	10–40 days	Mathialagan et al., 2017

**Table 4:** Reduction of ammonia volatilization, leaching, and nitrous oxide emissions by coated urea.

**Control release urea**	**NH_3_ volatilization %**	**N_2_O emissions %**	**NO${}_{3}^{-}$N leaching %**	**References**
Neem-Coated urea	27.5	12	18.3	Jadon et al., 2018
Sulfur-Coated urea	50	21	9.3	Hamadallah et al., 1988
Zinc-Coated urea	38	30	35.1	Kundu et al., 2016
Boron-Coated urea	25.6	17	3.2	Shivay et al., 2016
Carbon-Coated urea	27	24	33	Dawar et al., 2021
Polymer-Coated urea	62	45	58	Ullah et al., 2019
DMPP-Coated urea	41.1	50	20	Yang et al., 2016
NBPT-Coated urea	13.35	62	25	Emerson et al., 2021

**Table 5:** Application of coated urea to improve nitrogen use efficiency and yield of particular crops with specific coating %.

**Source of urea coating**	**Crop**	**Coating %**	**Increase in NUE %**	**Increase in yield %**	**References**
Neem oil-coated urea	Maize	3	63	75	Ali et al., 2020
Sulfur-Coated urea	Wheat	5	82	68	Shivay et al., 2019
Zinc-Coated urea	Rice	2	25	41	Shivay et al., 2019
Biochar-Coated urea	Rapeseed	1.5	58	16	Liao et al., 2020
Boron-Coated urea	Maize	0.5	42	75	Shivay et al., 2019
Polymer-Coated urea	Rice	6	78	26	Wang et al., 2015
Castor oil-coated urea	Maize	1	36	40	Shilpha et al., 2017
Agrotain-Coated urea	Wheat	3	38	37	Khan et al., 2013

## Factors affecting nutrient-coated urea and their availability

**Figure 3:**
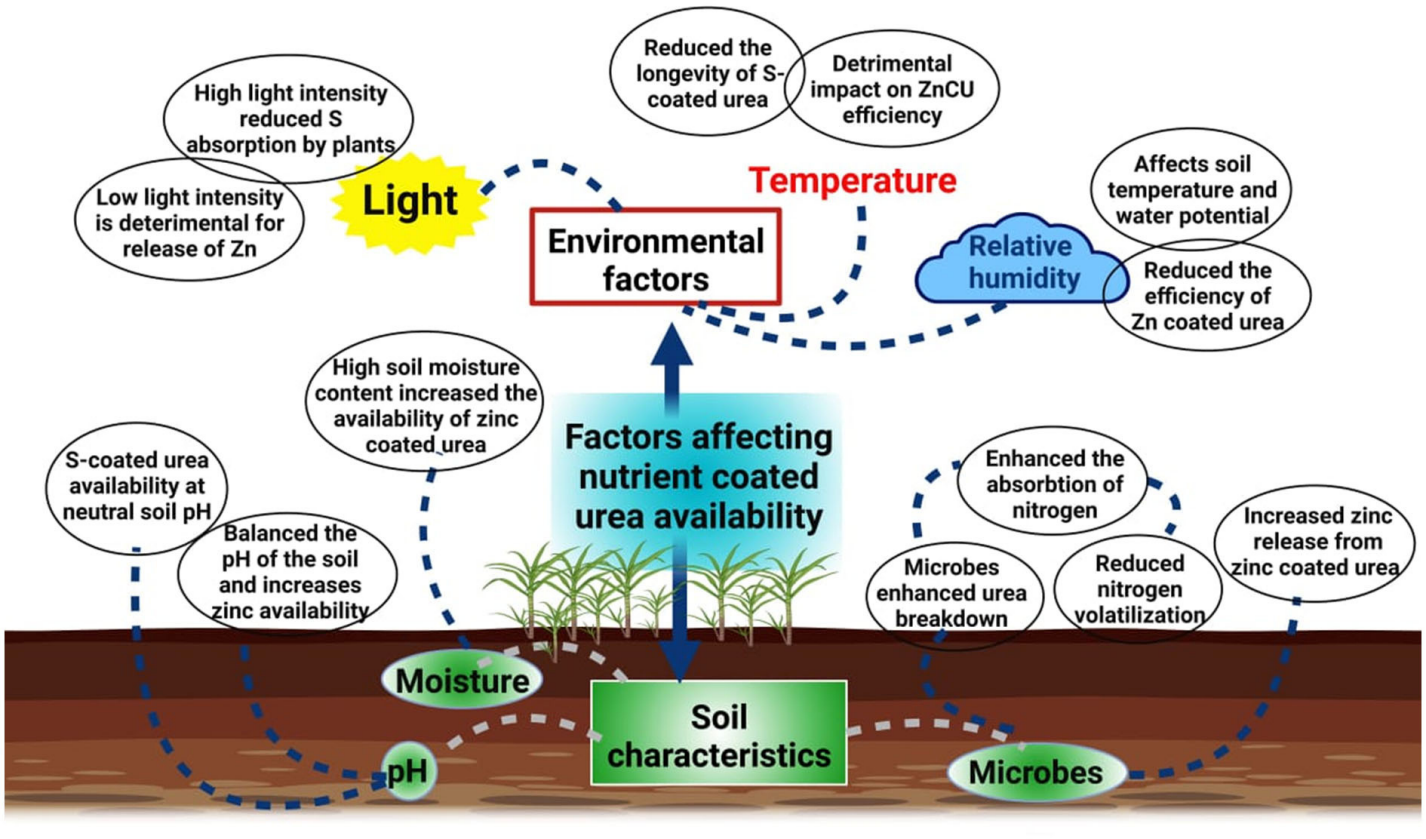
Factors affecting the availability of coated urea in soil. Low temperatures, moisture content, and low light intensity reduces the availability of coated urea in soil.

Soil pH, alkalinity, poor soil organic matter (OM), water holding capacity of the soil, microbial activity, amount and forms of minerals in the soil, soil moisture content, and soil texture all influence the availability of nutrients to plants, particularly zinc and sulfur (Figure 3). Other parameters that impact the potential of zinc and sulfur-coated urea include temperature, humidity, and light intensity, as well as interactions among other nutrients.

## Effect of soil pH on coated urea

Soil pH greatly influences the availability of plant nutrients by changing their forms. Low pH alleviates the macro and secondary macronutrients, however high pH reduces the availability of some micronutrients. Microbial activity may be diminished or altered in varying soil pH (Neina, 2019) as soil pH triggers the abundance and movement of S oxidizers in the soil (Zhao et al., 2015). The potential of S-coated urea is more pronounced in neutral soil pH and plants cannot uptake sulfur at lower soil pH. When sulfur is applied to the soil with low pH, the acidulated fertilizer lowers the percentage of nitrified N, which slows down the nitrification process and eventually stops as the pH drops below six. Urease activity picks up as the pH of the soil rises, peaking at a pH of 6.0–6.5 (Beig et al., 2020). The only cost-effective way to lower pH in the rhizosphere is to apply sulfur-coated urea to the soil (Akay et al., 2019). Soil pH also controls zinc mobility in soil (Sauvé et al., 2000; Smolders et al., 2004). Zinc solubility in soil declines 100-fold for each pH unit rise in soil and Zn availability reduces in high soil pH (Zhao et al., 2016). Rengel (2015) stated that the soil pH (more than 6.5) significantly reduces Zn availability to plants. A high deficiency of Zn was found in sandy soils compared to clayey soils (Suganya et al., 2020), though clay particles reduce the Zn uptake (Moreno-Lora and Delgado, 2020). In addition, Ain et al. (2020) reported that calcium carbonate concentration due to high Ca^2+^ ions in elevated soil pH fixes Zn in soil colloids that make Zn unavailable to the plants. Thus, plants uptake less Zn in calcareous soils (Iratkar et al., 2014). However, Moosa et al. (2021) reported that the exchangeability of zinc isotopes increases with increasing pH in the soil. In another study, zinc uptake is more in soil having high OM (Iratkar et al., 2014). The breakdown of soil organic matter, nutrient cycling, and efficiency of micro-fauna is reducibly observed in acidic soil by Madigan et al. (2010). Low total Zn concentration, high pH and calcite concentration, and organic matter content are the key factors that affect Zn availability in plants (Kihara et al., 2020). The quantity of Zn adsorption and desorption is strongly linked to soil pH (Bereket, 2018). Low pH inhibits zinc adsorption more in sandy soils than in soils rich in colloidal-size components (Moosa et al., 2021). Zinc-coated urea decreases the pH of the soil, thus, it increases zinc availability in the rhizosphere. Babar et al. (2018) observed the positive effect of Zn-coated urea in controlling the fluctuation of pH ranging from 6.0 to 6.5 in submerged paddy soils. Hence, Zn-coated urea as a mineral-coated fertilizer significantly maintains the soil pH around the optimal plant value of 6.5.

## Effect of soil moisture content on availability of coated urea

The effect of soil water potential on the degradation of coating components, urea absorption, and/or diffusion of dissolved urea from the granules may alter the rate of urea release from SCU (Zhang et al., 2011). The rate of nitrogen release from SCU depends on the type of coating, coating thickness, soil moisture, and soil temperature (Halvorson et al., 2014), whereas water permeability alters the nutrient release dynamic of the coated urea (Irfan et al., 2018). The coated material in SCU expands as it comes into contact with the soil water and gets converted into a hydrogel. Water diffuses into the cross-linked coated network, allowing the soluble half of the fertilizer to be gently released into the soil (Remya et al., 2021).

High soil moisture content improves the availability of zinc-coated urea in the soil (Angle et al., 2003). The amount of nutrients released is related to the water vapor pressure and is regulated by the ion concentration of the liquid medium. The moisture found in lower layers of the soil develops a moisture gradient, which augments the soil microbial activities responsible for nitrification and denitrification processes of urea in the soil (Kuang et al., 2019). In addition, the highest nitrification process was observed with 60% pore spaces of soil filled with water (Jinuntuya-Nortman et al., 2008). While soil moisture has a substantial impact on the rate of nitrogen release from Zn-coated urea, more nitrogen is released when soil humidity rises (Irfan et al., 2018). The rate of nutrient release is proportional to soil water pressure. The influence of soil moisture on nutrient release patterns may be described by the effects of vapor migration; however, this does not appear to be consistent with a diffusion process in the liquid phase (Moosa et al., 2021).

## Effect of microbial activity on availability of coated urea

Microbial activity in the soil has been identified as a key component influencing nitrogen (N) release from slow-release fertilizers. The N released from S-coated urea is mainly governed by the activity of soil microbes (Nardi et al., 2018). In addition, nitrogen release can be delayed by 6–8 weeks by reducing soil microbial populations (Nardi et al., 2018). Sulfur-coated urea is hydrolyzed by microbial ureases into ammonia and carbon dioxide (Rutherford, 2014). Microbes cannot perform efficiently in low soil water as their potential is reduced; therefore, nitrogen release from SCU is reduced (Moosa et al., 2021). Moreover, when the concentration of urea around soil microsites and soil pH increases due to the application of urea fertilizer, urease enzyme activity starts to hydrolyze the applied urea which eventually causes N losses (Watson, 2000; Krajewska, 2009). The SCU acts as a urease inhibitor, a possible N loss remedy because it inhibits the activity of the urease enzyme in the soil and reduces the rate of hydrolysis of urea in soil (Shaviv, 2001; Bolan et al., 2004).

Microorganisms in the soil play a significant role in the release of nitrogen in the soil (Morgan et al., 2009). The release pattern of coated urea is directly proportional to the microbial activity in soil (Gil-Ortiz et al., 2020). Soil microbes cause complete urea breakdown, allowing plants to absorb nitrogen, reducing nitrogen volatilization, and increasing zinc release from zinc-coated urea (Giroto et al., 2021). Bacteria convert nitrogen in the compound to nitrate, lowering soil pH and diminishing soil microbial diversity, which has a detrimental influence on the coated urea release pattern with zinc nanoparticles (Sadiq et al., 2021). Soil bacteria infiltrate the coating through fractures in the material and break down the urea granules; the microbial population alters the nutrient release and duration of zinc-coated urea fertilizer (Santos et al., 2018). The rate of breakdown of coated urea fertilizers containing Zn is mostly determined by soil temperature and density of soil microflora, and the duration is also determined by the activity of soil microbes (Yang et al., 2018). Zinc-coated urea reduces the rate of urea hydrolysis and serves as a urease inhibitor which increases crop growth and yield (Babar et al., 2018).

## Environmental factors

Nitrogen is found in the form of ammonia (NH_3_), nitrous oxide (N_2_O), and nitrate (NO${}_{3}^{-}$) in the atmosphere. Nutrient-coated urea is one of the most effective ways to reduce environmental losses while also providing other nutrients that are coated on the urea to have a synergetic effect on the urea. Many environmental conditions, including light intensity, humidity, and temperature, have an impact on the dynamics of nutrient-coated urea. These environmental conditions affect nutrient requirements and availability in plants, and they are listed below:

## Effect of light intensity on availability of coated urea

Sulfur concentration is badly affected by a high light intensity that reduces the plant biomass and accumulation of sulfur (Zenda et al., 2021). The ideal average light intensity is 30–40 micro Em^−2^, which does not deplete the concentration of sulfur. Higher light intensity boosts PSII activity, resulting in increased hydrogen production and a reduction in S concentration (Tatyana et al., 2004). Photo-degradation of SCU depends on the intensity and type of light (Gafoor et al., 2021). Light intensity has no direct effect on SCU efficiency, but it does reduce S absorption by plants, which has a negative impact on plant growth, quality, and NUE. Variations in light intensity may influence the rate of diffusion of SCU (Adams et al., 2013).

In contrast, zinc concentrations in plants are improved by high light intensity up to a certain point. As the light intensity rises from 200 to 3,000–4,000 foot-candle, zinc responses are enhanced; any further increase up to 11,000 foot-candle decreases zinc response (Baligar et al., 2006). When light intensity reaches near saturation point in photosynthesis, Zn reaction is at its peak. Due to Zn deficiency in crops, low light intensity limits root and shoot development (Edwards and Kamprath, 1974). Zinc-coated urea enhances pecan (a fruit having a single stone) growth and development by increasing photosynthesis and catching greater light intensity. Although low light intensity does not influence the release mechanism of Zn-coated urea, Zn absorption in plants is limited, which has a detrimental impact on plant development and production (Sadiq et al., 2021).

## Effect of relative humidity on availability of coated urea

In general, the release of coated fertilizer increases with increasing soil humidity. Even if the soil moisture is below field capacity, the osmotic potential of high humidity in soil augments the release of dissolved nitrogen from coated material to soil media (Christianson, 1988). However, sulfur metabolite absorption is susceptible to high humidity that inhibits sulfate uptake and severely impairs photosynthesis in plants. Low vapor pressure deficit (VPD) affects the stomatal conductance, reduces S absorption, and thus, photosynthetic efficiency is lowered. Shivay et al. (2016) observed that the diffusion rate is maximum in low field capacity soil moisture. Humid conditions are required for the disintegration and transport of nitrogen from coated urea fertilizers outward as humidity lengthens the duration of SCU and alters the coating material's release process (Baboo and Manager, 2016).

Relative humidity reduces zinc concentrations in plants and limits the availability of other micronutrients (Cu, Fe) in green tea shoots (Sud et al., 1995). For instance, Muster et al. (2011) reported that Zn uptake is greatly affected by high temperatures in humid environments. High humidity contributes to accelerating the release of nitrogen from Zn-coated urea by increasing the amount of water retained at the soil surface (Giroto et al., 2021). Humidity affects soil temperature and water potential, reducing the efficiency of Zn-coated urea in soil and making it easier to solubilize the urea granules by adding water through coatings (Sadiq et al., 2021).

## Effect of temperature on availability of coated urea

High temperature impairs photosynthesis, respiration, reproduction, and pollen development. Sulfur-coated urea is an emerging and efficient method to enhance plant defense mechanisms under heat stress conditions. Sulfur biomolecules ameliorate many negative impacts of environmental factors in plants (Ihsan et al., 2019). Nitrogen release from SCU fertilizers as diffusion rates is considerably boosted when sufficient moisture is available, moreover, its release is temperature dependent (Du et al., 2006; Morgan et al., 2009). For instance, high temperatures result in the early release of N from CRU fertilizer application in soil (Christianson, 1988). Similarly, Shivay et al. (2016) observed that temperature extremes reduced the longevity of all SCU products by altering the release mechanism of SCU along with soil characteristics such as soil moisture, temperature, and microbial activity.

Zinc intake and availability are restricted at high temperatures (Han et al., 2019), affecting chloroplast ultrastructure, its concentration, and chlorophyll fluorescence in zinc-deficient plants (Peck and McDonald, 2010). The use of nitrogen in combination with zinc, such as zinc-coated urea, minimizes the negative effects of high temperatures in plants to a certain extent (Raj et al., 2015). Heat stress in plants may be alleviated by providing Zn through Zn-coated urea, which boosts Zn levels in various plant sections; Zn increases ABA concentration, which in turn, strengthens the plant's defense system (Nazir et al., 2021). The kinetics of Zn-coated urea in the soil is altered by high temperatures, which has a detrimental impact on ZnCU efficiency (Nazir et al., 2021).

To minimize N loss in changing environmental conditions, controlled/slow-release fertilizers are specially designed that can sufficiently regulate the N requirement of crops during the growing season (Janke et al., 2022).

## Interaction with other nutrients affects the availability of coated urea

The interaction between two or more nutrients could have a synergistic (positive), antagonistic (negative), or no influence on the availability and crop uptake of other nutrients but mostly interactions among the macronutrients represent synergistic results that enhance crop NUE (Aulakh and Malhi, 2004; Rietra et al., 2017). Likewise, studies found that the macronutrient K is an essential nutrient like S and Zn required for crop growth and development and could have a synergistic effect with S and Zn availability (Jones and Jacobsen, 2005).

Sulfur has an antagonistic relationship with potassium; hence potassium buildup depletes the sulfur content of plants. Sulfur should be used in conjunction with NPK, which is designed to provide agronomic bio-fortification (Klikocka and Marks, 2018). Sulfur and nitrogen have a synergetic effect, and because both nutrients are utilized in protein synthesis, a precise N:S ratio increases crop quality and production. At various levels, several interactions were discovered in the absorption of NO_3_ and SO_4_ in plants (Klikocka and Cybulska, 2014). Molybdenum molecules are comparable in size to sulfur and their concentration significantly increases in the presence of sulfur, while White et al. (2004) observed selenium replaces sulfur molecules in plant biology. Sulfur increases the content and availability of micronutrients such as Fe, Mn, Zn, and Cu. In high soil pH circumstances that limit sulfur concentrations or where sulfur contents are low, it is advisable to add sulfur-containing fertilizers or sulfur-coated urea (Klikocka and Marks, 2018). Zinc has a positive relationship with nitrogen and potassium, but antagonistic relationships with phosphorus, iron, calcium, and copper. They have a detrimental impact because these nutrients obstruct zinc absorption and transfer from roots to shoots in plants. For instance, Ca and Zn fight for adsorption sites on the soil surface and in the roots (Irfan et al., 2018). Mycorrhizal infection of roots was suppressed when P was increased by lowering zinc levels. Plants' zinc requirements are harmed by increased phosphorus deposition in their leaves (Shivay et al., 2008). Zinc-coated urea is a way to protect plants from boron toxicity (Irfan et al., 2018) and Zinc insufficiency boosts Fe and Mn concentrations in wet soils while zinc-copper interaction increases wheat production (Shivay et al., 2008).

## Enhancement of yield through the application of nutrient-coated urea

Urea is a common fertilizer that is highly soluble in water and has volatilization as well as leaching losses severely impacting farmers' costs (Shivay et al., 2016). Urea granules should be encapsulated with less soluble compounds to avoid this problem. In addition, urea can be coated with sulfur, urease inhibitors, and other biodegradable environmentally safe compounds (Khan et al., 2015). Agricultural productivity is partly attributed to a continuous and long-lasting delivery of nutrients, which can only be achieved by using nutrient-coated urea. The use of SCU delays the nitrogen release while enhancing its utilization efficiency in rice fields. Rice production increased up to 55–68% with the use of sulfur-coated urea. The use of SCU in wheat and flooded rice fields minimizes nitrogen leaching losses (Wei et al., 2020). According to Shivay et al. (2019), the yield and seasonal distribution of growth achieved with a single application of sulfur-coated urea were similar to those obtained with repeated (3–5) applications of urea. Increased plant usage efficiency due to extended soil retention and fewer fertilizer applications, and resultant fertilizer and application cost savings are prospective benefits of using coated urea (Nasima et al., 2010). Sulfur-coated urea increased dry matter production in rice, nitrogen absorption was increased up to 39.4%, and grain protein percentage was boosted up to 5.8–14.9% with the use of SCU as compared to normal urea application in rice (Khan et al., 2015).

Zinc-coated urea increased grain yield by around 28% and is quite effective toward plant physiological attributes (Nazir et al., 2021). As compared to prilled urea, 29% of rice production was increased by the use of zinc-coated urea (Shivay et al., 2019). Zinc enhances several biochemical, physiological, quality, and yield-related factors in cereals by restoring plant vigor, oil content, sugars, and pigments. Using Zn-coated urea to slow down the hydrolysis and nitrification process resulted in enhanced N absorption and, as a result, a higher yield (Affendi et al., 2018). Zinc-coated urea also increased the yield by improving NUE while completing the Zn need of the sunflower crop (Sadiq et al., 2021). The application of zinc-coated urea to rice resulted in an increased transpiration rate and a higher percentage of normal kernels while lowering the percentage of opaque, abortive, and chalky kernels (Irshad et al., 2021). In comparison to the prilled urea (PU), the application of Zn-coated urea increased the yield by 29% and Zn concentration in aromatic rice grain (Shivay et al., 2019). As a result, the Zn concentration in zinc-coated urea has a significant impact on crop quality and output (Suganya et al., 2020). Zn content in maize grain was much greater than PU, and with 2.5 percent of ZnCU, total Zn intake (grain + stover) increased by 32.4 percent and improved crop yield (Pooniya et al., 2018). In maize, total zinc intake was 32.4% higher with the use of 2.5% Zn-coated urea compared to un-coated fertilizer, which increased the yield by about just 4.6% (Pooniya et al., 2018).

## Enhancement of grain quality through the application of nutrient-coated urea

In comparison to prilled urea, sulfur-coated urea enhanced nitrogen absorption by 39.4% and increased grain protein percentage from 5.8 to 149%. Furthermore, S was active in the production of proteins, chlorophyll content, vitamins, and sulfur-containing amino acids like cysteine and methionine, which are essential components of protein (Naiknaware et al., 2015). Coating urea with sulfur resulted in more N remaining in the soil for longer periods. A higher proportion of absorbed N, and more N being translocated into the grain, improved urea N efficiency and grain quality (Khan et al., 2015). Sulfur-coated urea increased kernel size, density, oil content, protein content, and starch content while decreasing virtuousness, damaged kernels, and fungal infections, all of which contributed to grain quality (Haseeb-ur-Rehman et al., 2022).

Zinc-coated urea boosts Zn levels in a variety of plant components, including leaves, tubers, and fruit, as well as the whole grain and endosperm (Wakeel et al., 2018). To acquire good quality rice (increased Zn concentration in rice grains), special attention should be paid to balancing Zn levels in soil, which can only be achieved by using Zn-coated urea (Amanullah and Inamullah, 2016). Zn-coated urea improves Zn absorption and partitioning into different plant parts, potentially increasing rice grain output and nutritional quality (Wang et al., 2014). It also aids in the absorption of zinc in grains, which helps to improve grain quality by boosting protein and amino acid content (Li et al., 2015).

## Enhancement of nitrogen use efficiency through the application of nutrient-coated urea

**Figure 4:**
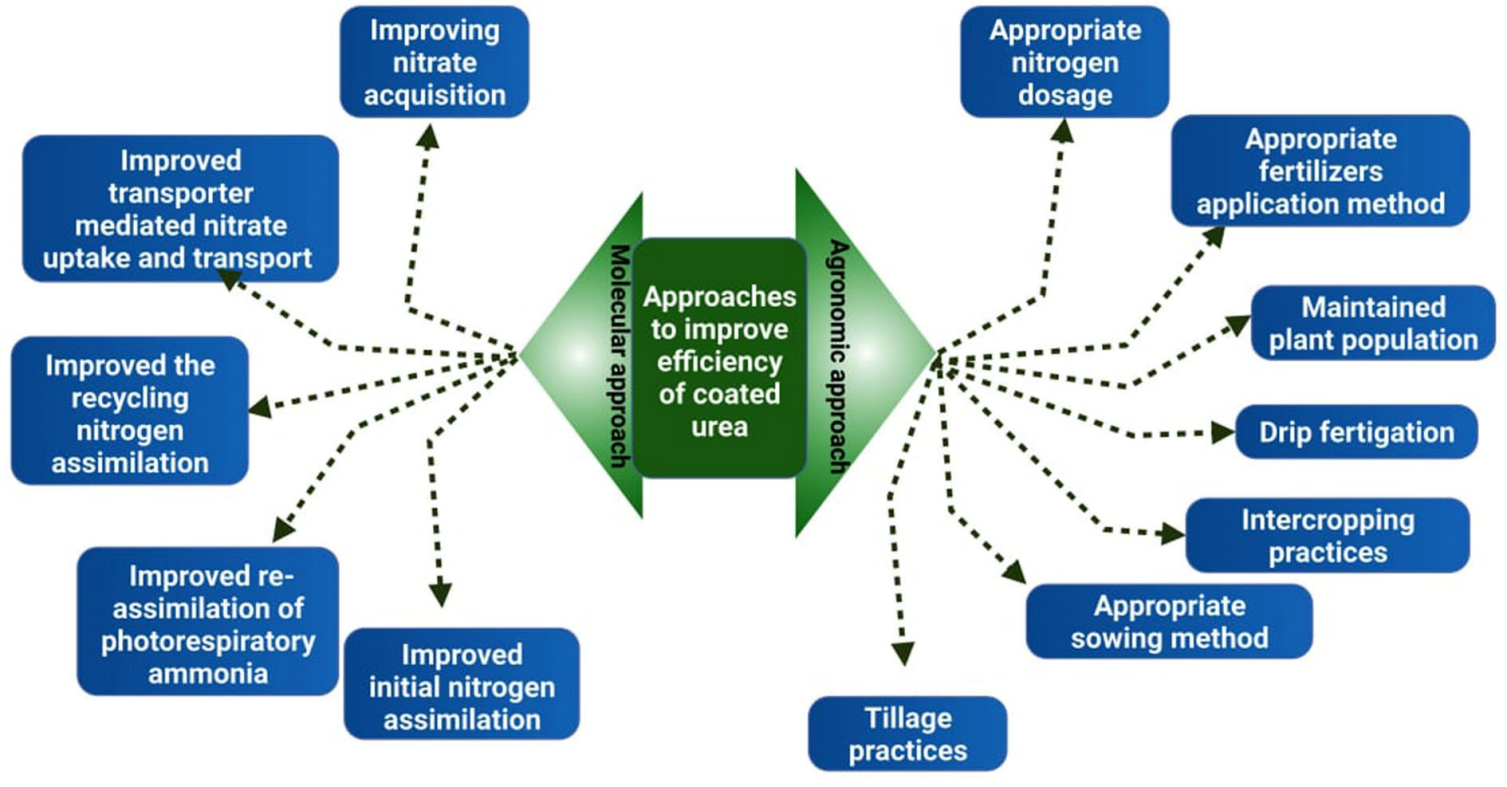
Approaches to improve the efficiency of coated urea. Appropriate dosage of nitrogen fertilizer, optimum plant population, drip fertigation, intercropping, appropriate sowing method, and improved genetic makeup of plants can enhance the utilization efficiency of coated urea.

Coated urea fertilizers increase nitrogen supply while lowering nitrogen losses in the form of leaching, volatilization, and N_2_O emission (Jadon et al., 2018; Wei et al., 2020). Normal urea is less efficient as compared to nutrient-coated urea whose NUE (Figure 4) is 30–60% less than coated urea (Chen et al., 2020). With coating, 20–30% dose of urea can be saved than normal urea application while increasing its uptake and higher yield production. It increases nitrogen agronomy efficiency (NAE; 23.4%), reduces nitrogen fertilizer utilization rate (NUR; 34.65%), and enhances 25.83% nitrogen physiological efficiency (NPE; 25.83%) (Zhu et al., 2020).

The use of sulfur improves NUE by 50% and nitrogen recovery efficiency by 60% in wheat crops (Byatvarkeshi and Zareabbyaneh, 2016; Shivay et al., 2016). Additionally, volatilization losses of nitrogen are decreased in coated urea which improves nitrogen recovery in plants (Wang et al., 2016; Wei et al., 2020). For instance, Gafoor et al. (2021) found the least nitrate concentration (7.37 kg ha^−1^) with SCU owing to reduced nitrogen losses in groundwater table from wheat fields. With the application of zinc-coated urea, NO_3_-N leaching decreased to 35.1% (Jadon et al., 2018). Nitrogen use efficiency improved by up to 55% using Zn-coated urea, and ammonia volatilization losses were greatly reduced (Sadiq et al., 2021). Zn-coated urea enhanced NUE by up to 80%, increased rice yield by 50%, and grain Zn content by 126%. It provides nutrients to plants in a regulated and delayed way, reducing nutrient emissions, leaching, and runoff losses, while simultaneously improving crop NUE and yield (Sadiq et al., 2021). Nitrogen losses can be reduced by using Zn-coated urea, and N efficiency can be raised by slowing down the rate of urea hydrolysis in the soil (Babar et al., 2016).

## Residual effects of nutrient-coated urea

Application of N fertilizer beyond its need generates undesirable consequences which result in the degradation of water, soil, and air quality. It also leads to soil acidification, decreased groundwater quality due to nitrate leaching, and increased nitrous oxide (N_2_O) emissions, which is a 300-fold more powerful greenhouse gas than CO_2_ contributing to global warming. When the concentration of nitrate exceeds 10 mg L^−1^ in drinking water, it becomes unfit for humans and animals (Jadon et al., 2018).

The accumulation of S in the soil is associated with reduced soil pH, inhibited root growth, and nutrient intake, as S is susceptible to leaching and the formation of sulfuric acid in soil media is common (Liu et al., 2010). Sulfur dioxide toxicity causes excessive water loss due to increased stomatal opening leading to water scarcity and thus disrupting photosynthetic efficiency and consequently reducing photosynthesis. In addition, S toxicity causes root damage, leaf senescence, and reduced growth of crop plants (Liu et al., 2010). Plant growth is impeded if soil S concentrations in the upper horizon (0–20 cm) reach 4% (Likus-Cieślik et al., 2017). The proficiency of Zn fertilizer rarely exceeds 10% because Zn is voluntarily fixed with soil colloids and is mostly available for succeeding crops due to its fixing nature (Li et al., 2013).

Zinc diffuses widely and reaches a phytotoxic level in many soils due to the intense application of excess fertilizers, pesticides, manures, sewage sludges, smelters, incinerators, mines, and galvanized products (Kaur and Garg, 2021). Zn toxicity causes curling and rolling of young leaves, stunting shoot growth, chlorosis, and death of leaf marginal cells. In high concentrations of Zn, cell organelles collapse leading to damage in the cytoplasmic structure in plants, and concentrations >10 mg/kg or 7.5 mM condenses the chromatic material, dilates the nuclear membrane, and disrupts the cortical cells (Moreira et al., 2018; Ning et al., 2020). Moreover, the intake of iron is inhibited by Zn toxicity in the soils, whereas Zn toxicity is caused by an overabundance of magnesium in the soil (Moreira et al., 2018).

## Constraints in adopting coated fertilizers

There are several constraints to using nutrient-coated urea: (i) lack of farmer awareness about the need for nutrient-coated urea fertilizers, especially S and Zn-coated urea. Farmers are unaware of the effectiveness and significance of coated urea fertilizers, which is the task of extension workers to go door to door to advise farmers that coated urea not only reduces the amount of application as well as costs without reducing yield, but also adds additional nutrients to the soil, increases soil health, and crop quality; (ii) lack of monetary access for coated fertilizers; (iii) the source of information is not reliable; (iv) lack of knowledge on making nutrient-coated urea; (v) lack of capital; (vi) difficulty in calculating the optimal dose of nutrients to coat urea; and (vii) not available on time (Chouhan et al., 2018).

## Agronomic approach to improving the efficiency of coated urea

The addition of organic manures to soils provides several micronutrients (Ca, Mg, S, Mn, Fe) that make it more fertile for better crop production by improving the soil's physical (sand, silt, and clay percentages), chemical (CEC, WHC, and pH), and biological (soil fauna population) properties (Wang et al., 2020). Crop rotation is the major agronomic practice that is adopted for sustainable nutrient balance in the soil. Nitrogen losses are too high, up to 70% of total available nitrogen, due to excess nitrogen, low plant population, inefficient application methods, or other factors. Improved agronomic practices such as appropriate nitrogen dosage, application of nitrogen through canopy sensors, maintaining plant population, drip fertigation, and legume-based intercropping can reduce these losses by up to 15–30% (Shivay et al., 2016). The amount of fertilizer applied is mostly dictated by soil properties and agroclimatic conditions. The improvement in nutrient utilization can be achieved using nutrient-coated fertilizer application in crop fields and that is the best agronomic approach to modern agriculture-farming (Luo et al., 2018). The coated material (sulfur and zinc) has a synergetic effect with urea that enhances nitrogen absorption and increases NUE (Shivay et al., 2016). Wang et al. (2020) suggested that the use of sulfur and zinc-coated urea is the best approach for farmers to alleviate sulfur and zinc deficiencies in crops. Bhuiyan (1994) found nutrient-coated urea better than poultry manure (PM) and it increased the efficiency of applied fertilizers. Sowing of crops on ridges and application of nutrient-coated urea fertilizer improves the water use efficiency of crops. Laser land leveling, conservation tillage and choosing good quality seeds, and sowing and irrigation methods along with coated urea fertilizers can change the whole scenario of agriculture and make crops vigorous and bio-fortified with specific nutrients whose availability is far less than their demand (Khan et al., 2006).

The use of nutrient-coated fertilizers (urea), therefore, is the straightforward and economically best approach that enhances food production to meet the current food security challenge. But it requires specific consideration in terms of nutrient source, application technique, and environmental consequences. Nutrient-coated urea must be administered as a single dose during each crop season, making them cost-effective in most situations.

## Molecular approach to improving the efficiency of coated urea

Molecular techniques refer to the genetic modification that is done at DNA molecular level to enhance desired traits in plants, and these tools and techniques should be considered for crop improvement. The tools contain molecular marker techniques like PCR amplified DNA sequences, RAPD, and AFLP. Researchers should choose the appropriate technique that is best suited for their respective programs and they should be encouraged to integrate molecular tools based on gene maps, gene cloning, QTL mapping, marker-assisted selection, microsatellites, SNP, and molecular cytogenetics. Molecular approaches facilitate monitoring of plant health, detecting pathogens, reducing disease spread, and aiding in better crop management (Wan et al., 2017). In this context, nitrogen uptake, assimilation, and utilization by plants through DNA markers are new emerging molecular techniques that help improve fertilizer use efficiency. The dynamic character of nitrogen, as well as its tendency to leach into the soil and environment, provides a unique challenge for its better management. The Nitrate Transporter 1/Peptide Transporter (NPF) family (Léran et al., 2014), the Nitrate Transporter 2 (NRT2) family, the Chloride Channel (CLC) family, the Slow Anion Associated Channel Homolog (SLC/SLAH) family, and aluminum-activated malate transporters (ALMT) family mediate nitrate uptake and transport in plants (Li H. et al., 2017). Many other metabolic processes and gene expression levels regulate nitrogen in plants. The reduction of nitrate to nitrite by the nitrate reductase enzyme happens in the cytosol. Nitrite is carried into chloroplasts in leaves and transformed into ammonium ions by the enzyme nitrite reductase. During the production of nucleic acid, chlorophyll, and amino acids, the products of ammonia, glutamine, and glutamate, serve as nitrogen donors. Three major ammonium assimilation processes have been proposed: initial nitrogen assimilation, reassimilation of photorespiratory ammonia, and “recycled” nitrogen assimilation. Organic nitrogen is transported from source to sink organs in the form of amino acids. Due to ammonium remobilization, ammonium absorbing enzymes are required during the grain filling stage (Wan et al., 2017). The GS and GOGAT pathways are involved in the production of amino acids from ammonia. Two types of genes, GLN1 and GLN2, are regulated by GS with a decameric structure. GLN2 (single nuclear gene) encodes chloroplastic GS2, which is involved in ammonium absorption or re-assimilation in C3 and C4 plants, or from the photorespiratory product of C3 plants. The GLN1 gene family, on the other hand, encodes the GS1 isoform, which recycles ammonium during leaf senescence and transport in phloem sap. Overexpression of HATS-like NRT2.1 enhances nitrate influx, while NUE and its phenotypic utilization remain unaltered. The transgenic rice plant's grain production improved by overexpressing NADH-GOGAT. To increase yield by overexpressing GS or GOGAT genes, it is necessary to understand the alleles of genes and their promoters (Zhou et al., 2017). In Arabidopsis, overexpression of ASN1 boosted soluble protein content in seed, total protein, and the plant's ability to grow on a nitrogen-limited diet. These findings indicate that modifying downstream N-remobilization stages could improve NUE (Li W. et al., 2017).

The inconsistency of overexpressed important enzymes (NR, NiR, GS, and GOGAT) for improving NUE or phenotypic change is also a problem. Nitrogen utilization efficiency is linked to nitrate acquisition, which can be improved further by modifying nitrate assimilation enzymes and proteins using various biotechnological techniques. To improve NUE, it is critical to target several mechanisms, enzymes, and variables rather than focusing on a single rate-limiting mechanism. As a result of these factors, new molecular techniques such as microarray and transcriptome are being considered emerging tools for studying the response of the entire genome of plants. In all plants, whole genome RNA sequencing is a current way of understanding changes at the genomic level, gene expression level, and individual genes associated with desirable features. In the future, the combination of DEGs (differentially expressed genes) with QTL databases will be critical in developing new nitrogen use-efficient genotypes (Gelli et al., 2014). It is concluded that a combination of agronomic and molecular techniques has the potential to improve nitrogen use efficiency.

## Conclusion and future thrusts

Nutrient-coated urea is an efficient N management approach for ensuring better productivity in crops, reducing nitrogen losses (leaching, volatilization, and denitrification), and the subsequent impact on the environment. The application of S and Zn-coated urea improved chloroplast structure, chlorophyll contents, stomatal conductance, photosynthetic and transpiration rate, assimilate partitioning, protein synthesis, and antioxidant activity, therefore improving plant growth, biomass, and grain yield. Moreover, S and Zn-coated urea substantially improved the NUE and reduced N losses as compared to conventional urea. Controlled release urea (CRU) or nutrient-coated urea is less soluble due to the coating on the urea granules that progressively releases the fertilizer making it ideal for crop synchronization. It retains the nutrients in the soil for the longest possible time and improves water use efficiency, soil water retention capacity, soil microbial activity, soil texture and structure, and ultimately soil quality. Environmental factors such as light intensity, humidity, and temperature also play an important role in the release mechanism of coated urea and its availability in soil for plants.

However, there are many unanswered questions. Future research should concentrate on molecular approaches such as marker-assisted selection and inclusive hybridization (integration of desirable genes), as well as biotechnological approaches, such as next-generation DNA and RNA sequencing, and genetic alterations to create genotypes with improved zinc and sulfur contents and aptitudes to acquire more sulfur and zinc from the soil. A GIS-based mapping can be used to discover nutrient-deficient zones/regions to regulate the most appropriate basis of nutrient-coated fertilizer and application methods to boost nutrient efficiency. Broad and long-term research should be done on the release kinetics of nutrient-coated urea, particularly on how their release synchronizes with the requirement of the plant. The role of coated urea should be explored under modern sowing methods including bed and ridge sowing methods and new irrigation methods including drip and sprinkler irrigations. Its role in acid and highly alkaline soils should be studied as the soil pH is a major factor that affects nutrient uptake by plants. The effect of Zn and sulfur-coated urea on soil microbial activities and their compositions are poorly studied, and it is imperative to bridge this research gap. Similarly, the role of sulfur and Zn-coated urea in plant functioning is also poorly studied. For instance, the role of coated urea in germination mechanisms has not been explored and it would be pertinent to explore the role coated urea plays in seed germination. While the role of sulfur and Zn-coated urea on nutrient uptake has been well-researched, their role in nutrient signaling and their impact on nutrient and ionic transporters needs attention. Likewise, their influence on stomata movement, electron transport, and efficiency of PS-II is poorly studied, therefore it is important to explore their roles in these aspects. Additionally, their impact on seed quality compositions and genes involved in anti-oxidant activities needs further study. The effect of S and Zn-coated urea on different hormones and osmolytes have not been adequately explored, particularly under normal and diverse stress conditions. Lastly, their impact on plant growth, yield, and various physiological functions under different abiotic stress (cold, drought, heat, heavy metals, and salinity stress) must also be studied given that zinc, sulfur, and nitrogen play an appreciable role in plant tolerance against different stresses.

## Author contributions

AMu, IK, and MC: conceptualization. AMu, IK, MC, and MH: writing original draft. MN, AS, AMd, MB, MJ, NA, and RG: writing-review and editing. All authors have read and agreed to the published version of the manuscript.

## Conflict of interest

The authors declare that the research was conducted in the absence of any commercial or financial relationships that could be construed as a potential conflict of interest.

## Publisher's note

All claims expressed in this article are solely those of the authors and do not necessarily represent those of their affiliated organizations, or those of the publisher, the editors and the reviewers. Any product that may be evaluated in this article, or claim that may be made by its manufacturer, is not guaranteed or endorsed by the publisher.
